# Type IV collagen as a potential biomarker of metastatic breast cancer

**DOI:** 10.1007/s10585-021-10082-2

**Published:** 2021-03-03

**Authors:** Moa Lindgren, Malin Jansson, Björn Tavelin, Luc Dirix, Peter Vermeulen, Hanna Nyström

**Affiliations:** 1 Department of Surgery, Department of Surgical and Perioperative Sciences, Umeå University, By 10A, Norrlands Universitetssjukhus, Umeå, 901 85 Sweden; 2 Department of Radiation Sciences, Umeå University, Umeå, Sweden; 3 Translational Cancer Research Unit, GZA Hospital Sint Augustinus and Antwerp University, Antwerp, Belgium; 4 Wallenberg Centre for Molecular Medicine, Umeå University, Umeå, Sweden

**Keywords:** Breast cancer, Metastases, Biomarkers, Collagen IV, CA 15-3

## Abstract

No reliable, non-invasive biomarker of metastatic breast cancer (mBC) exists: circulating CA15-3 (cCA15-3) is the marker mostly used to monitor mBC. Circulating collagen IV (cCOLIV) has been evaluated in other metastatic cancers and has been found to be a promising biomarker. The overarching aim of this study was to evaluate cCOLIV as a potential biomarker in patients with mBC. The first aim was to determine the levels of cCOL IV and cCA15-3 in patients with healthy controls, primary breast cancer (pBC) and mBC. The second aim was to compare levels of cCOLIV and cCA15-3 in patients with different metastatic sites of BC. The third aim was to investigate the prognostic value of cCOLIV and cCA15-3 for mBC patients. The fourth aim was to analyse whether a combination of the two biomarkers was more accurate in detecting mBC than a single marker. Lastly, we investigated the tissue expression levels of COLIV in BC bone metastases (BM) and liver metastases (LM). Plasma levels of cCOLIV and cCA15-3 from healthy controls and patients with pBC and mBC were measured. COLIV expression in tissue from patients with LM and BM was analysed using immunohistochemistry. Clinical and survival data were collected from medical charts. The levels of cCOLIV and cCA15-3 were significantly elevated in mBC patients compared with healthy controls and pBC patients. No differences in cCOLIV and cCA15-3 levels were found based on the metastatic site. High levels of cCOLIV, but not cCA15-3, correlated with poorer survival. cCOLIV alone and the combination of cCA15-3 and cCOLIV were superior to cCA15-3 at detecting mBC. COL IV was highly expressed in the tissue of LM and BM. Our study suggests that cCOLIV is a potential marker to monitor patients with BC.

**Supplementary Information:**

The online version of this article (10.1007/s10585-021-10082-2) contains supplementary material, which is available to authorized users.

## Introduction

More than 1.5 million women are diagnosed with breast cancer (BC) every year and it is the most common cancer among women [1–3]. In the western world, the prognosis for these patients has steadily improved over the last few decades, with an average five-year overall survival of 90% [4]. BC-associated mortality is mostly related to late diagnosis and metastatic spread and it is currently estimated that, each year, 500,000 deaths occur worldwide due to metastatic BC (mBC) [3].

Patients with a history of primary BC (pBC) are monitored by clinical examination and imaging to detect metastatic spread [4]. Personalised treatment is essential for patients with BC. Treatment is in fact guided by prognostic and predictive biomarkers, such as the tissue expression of oestrogen receptor (ER), progesterone receptor (PR) and human epidermal growth factor receptor 2 (HER2) [4, 5]. The diagnosis and treatment planning thus rely on invasive procedures and expensive medical imaging. In this context, markers in the blood could be of great use and are highly desirable.

Currently, no optimal non-invasive biomarker is available for either pBC or mBC. To our knowledge, MUC1-derived cancer antigen 15-3 (CA 15-3) is the only circulating biomarker used in the clinic. CA 15-3 is derived from tumour cells (TC) and is only used for monitoring advanced disease when standard imaging is unavailable [4, 6]. The limited applicability of CA 15-3 in explained by its low sensitivity and specificity. High circulating levels are present in patients with both pBC and mBC but also in patients with liver, ovarian and lung cancer and some benign conditions as well [7, 8]. In addition, not all mBC patients have high cCA 15-3 and the reported accuracy is highly dependent on the study set-up [9–14]. One study used a fixed cut-off value of cCA 15-3 (> 40 IU/l) to detect recurrent BC, either local or distant, which resulted in a sensitivity of 36% and a specificity of 97% [15]. In contrast, another study regarded an individual increase in cCA 15-3 of 100% as a strong indicator of metastatic disease and reported a sensitivity of 55.6% and a specificity of 99.8%. It also reported an increase in sensitivity when cCA 15-3 was used in combination with circulating carcinoembryonic antigen (cCEA) compared with when used alone [10]. Furthermore, cCA 15-3 appears to correlate with disease progression, tumour burden and the site of metastasis. It is reported that mBC patients with bone metastases (BM) and liver metastases (LM) present with higher levels of cCA 15-3, compared with lung and lymph node metastases [11, 16]. To conclude, the results show that cCA 15-3 is able to detect BC metastases in some patients but not in all [11].

The ideal biomarker of mBC should have a diagnostic, predictive and prognostic value and should be able to replace other monitoring procedures, including imaging. A biomarker of this kind would be of great clinical significance. The majority of currently available biomarkers in cancer are based on analytes derived from the tumour cells. A source of potential new biomarkers is the tumour stroma.

The stroma includes the non-cancerous cells, such as immune cells, vascular cells and fibroblasts, as well as extracellular matrix (ECM) proteins [17]. During tumour progression, the ECM of the stroma is the subject of constant remodelling, contributing to the increased deposition of ECM proteins in the tissue and in the circulation [18]. ECM proteins are potential candidates in the search for new circulating biomarkers. Since a tumour consists of both tumour cells and stroma, a combination of markers representing both compartments could potentially help to improve the sensitivity and specificity of a cancer biomarker. One promising ECM-related biomarker is the basement membrane protein, type IV collagen.

Circulating type IV collagen (cCOL IV) is a promising biomarker of other metastatic cancers. Colorectal cancer (CRC) patients with liver metastases (CLM) have higher levels of cCOL IV, compared with patients without metastases, and the level appears to coincide with tumour burden and a poorer prognosis [19, 20]. Furthermore, cCOL IV appears to be superior to the conventional biomarker, circulating carcinoembryonic antigen (cCEA), at identifying CRC patients with a poor prognosis [20] and cCOL IV in combination with cCEA is better at detecting CLM than either marker alone [21]. Even pBC patients have increased levels of cCOL IV compared with healthy controls and patients with a poor prognosis have high levels of cCOL IV in both serum and tissue [22, 23]. In addition, the development and progression of BC correlate with the increased expression of the α1-chain of COL IV (Col4A1) and an enzyme necessary for the 3D formation of COL IV in tissue (collagen prolyl-4-hydroxylase α isoform, P4HA2) [23]. This indicates that COL IV deposition may be important for BC metastases and that COL IV could potentially be used as a biomarker of mBC. To our knowledge, there are no published studies in which cCOL IV has been evaluated as a biomarker in patients with mBC.

The overall aim of this paper was to evaluate cCOL IV as a potential biomarker in patients with mBC. The specific aims are as follows. The first aim was to determine the levels of cCOL IV and cCA 15-3 in healthy controls and in patients with pBC and mBC. The second aim was to compare the levels of cCOL IV and CA 15-3 in patients with different metastatic sites of BC. The third aim was to investigate the potential prognostic value of cCOL IV and cCA15-3 for mBC patients. The fourth aim was to analyse whether a combination of the two biomarkers was more accurate in detecting mBC than a single marker. Lastly, we investigated the tissue expression levels of COL IV in bone metastases (BM) and liver metastases (LM) of patients with breast cancer.

## Materials and methods

### Patients and sample collection: plasma samples

In this study, cCOL IV and cCA15-3 were analysed in plasma samples from 44 female patients with mBC and in 48 female patients with pBC. As a control group, plasma samples from nine healthy women with no malignant history were analysed.

For the mBC cohort, patients with LM (n = 5), BM (n = 26) and patients with both liver and bone metastases (n = 13) were included. These metastatic patients were admitted to GZA Hospital Sint Augustinus, Antwerp, Belgium, and they were diagnosed as palliative between 2003 and 2013. The inclusion criteria were as follows: available plasma samples in female patients with diagnosed mBC; samples were collected at the start of treatment and, at the time of sampling, the exact metastatic sites were known from CT scans and bone scintigraphs.

For the pBC cohort, 48 female patients with primary breast cancer without distant metastases were included. The 48 patients were admitted for elective surgery to Umeå University Hospital, Sweden, during 2008–2010 and the selection of the patients was made to obtain an equal distribution of invasive ductal breast cancer with and without ductal cancer in situ (DCIS), node-positive and node-negative, HER2-positive and HER2-negative, hormone receptor-positive and receptor-negative tumours. The St Gallen International Expert Consensus guidelines [24] were used to subgroup the tumours according to the molecular subtype of the tumour and, when the marker ki-67 was missing, the histopathological grade was used to distinguish luminal A from B tumours. Furthermore, the inclusion criteria were available preoperative plasma samples collected four weeks before surgery and the absence of neoadjuvant radio- or chemotherapy.

The control cohort consisted of nine female patients admitted to Umeå University Hospital, Sweden. These patients underwent surgery for non-malignant breast disease during 2008–2010. None of these patients had any malignant disease.

All the plasma samples were frozen and stored at -80 °C until analysis. Clinical data were collected from patients’ charts.

### Patients and sample collection – tissue samples

COL IV protein expression in LM was assessed in 29 paraffin blocks from a total of 21 BC patients undergoing elective liver surgery at GZA Hospital Sint Augustinus, Antwerp, Belgium, in 2002–2012. Additionally, COL IV expression was assessed in 13 BM paraffin blocks from a total of 11 patients undergoing acute orthopaedic surgery for breast cancer bone metastases at Umeå University Hospital in 2008–2019.

### Analysis for measurements of circulating type IV collagen and CA 15-3

Plasma levels of cCOL IV were measured with a commercially available COL IV ELISA kit (sensitivity 0.1 ng/ml, Argutus Medical, Dublin, Ireland), a sandwich-ELISA kit with two monoclonal antibodies directed against the 7S and collagenous domain of COL IV. The manufacturers’ protocol was followed and the samples were analysed in duplicate, where the difference between samples was not allowed to be more than 12.5%. This ELISA kit was originally developed for measurements of serum samples but has been validated and used for the measurement of cCOL IV in plasma samples [19, 21, 25, 26].

Plasma levels of cCA 15-3 were analysed by an accredited national laboratory (Laboratoriemedicin, Sundsvall Hospital, Sweden), where cCA 15-3 was measured using an electrochemiluminescence immunoassay using Cobas 8000 (system name: c8000 17G4-07) (Roche, Basel, Switzerland). Samples were measured in duplicate.

### Immunohistochemistry

Five µm sections of paraffin-embedded samples were cut using a microtome (Leica RM 2165, Leica Microsystems, Wetzlar, Germany). All the sections were assessed by pathologists and confirmed as breast cancer metastases. Haematoxylin and eosin were used for routine staining. The immunohistochemical staining was performed with the Ventana Benchmark automated immunostainer (Ventana Medical Systems, Tucson, AZ, USA). As a primary antibody directed at human type IV collagen, the AB748 rabbit polyclonal was used at 1:50 and after protease pre-treatment for 30 min at 37 °C (Merck, Darmstadt, Germany). A secondary antibody linked to peroxidase followed by diaminobenzidine tetrahydrochloride (DAB) as a chromogen was used. As a negative control, the primary antibody was omitted. The expression intensity of COL IV in the metastases was graded as follows: 0 = no expression, 1 = mild to moderate expression and 2 = strong expression. This intensity scoring system for COL IV has been published earlier [21, 26]. The immunohistochemical scoring was performed by HN and ML.

### Statistical analysis

Kruskal–Wallis tests were applied to investigate the statistical significance of the differences in cCOL IV and cCA 15-3 between the cohorts, between the types of BC metastasis and when comparing patient characteristics between cohorts. The Mann–Whitney U test was used to estimate the statistical significance of the differences in cCOL IV and cCA 15-3 between control patients and pBC patients, between metastatic versus non-metastatic patients and between patients with two different types of metastatic site. The prognostic value of cCOL IV and cCA 15-3 was analysed with Kaplan–Meier analyses. The metastatic cohort was split at the median into two groups with low and high concentrations of cCOL IV and cCA 15-3. Survival was defined as the time between the date of sampling and the date of death from all causes. A log-rank test was used to test for differences in survival between groups and Cox regression analysis was used to analyse survival results adjusting for prognostic factors such as ER-, PR- status and number of organs involved. Receiver operating characteristic (ROC) analyses were applied to calculate the area under the curve (AUC) to establish whether cCA 15-3, cCOL IV, or a combination of both was better at detecting mBC. To find the optimal cut-off point for each marker we used the approach known as the point closest-to-(0,1) corner in the ROC plane which defines the optimal cut-point as the point minimizing the Euclidean distance between the ROC curve and the (0,1) point. In the ROC analysis and for calculating optimal cut-offs, the pBC and the mBC cohorts were analysed and metastatic disease was an event. SPSS version 25 (SPSS Inc, Chicago, US) and STATA version 15 (STATA Corp, TX, US) were used for the statistical analyses.

## Ethics

Informed consent was obtained from all patients and this study was approved by the national ethical review board of Sweden (EPN) and the ethical committee at the GZA Hospitals, Antwerp, Belgium.

## Results

### Characterisation of the plasma and tissue cohorts

Patient characteristics of the plasma cohorts of patients with pBC, mBC and non-malignant breast disease are presented in Tables 1, 2 and Supplementary Table 1. The median follow-up time for the pBC cohort and the control cohort was 10 years. The median follow-up time for the mBC cohort was 26.7 months. Ten patients in the pBC cohort developed distant metastasis and eight of them died of breast cancer-related events (16.7%) during this period. Three patients died of causes other than breast cancer (6.3%). In the mBC cohort, 36 patients died during the follow-up (81.8%).

**Table 1:** Patient characteristics of cohorts; healthy controls, primary breast cancer (pBC) and metastatic breast cancer (mBC) and values for circulating type IV collagen (cCOL IV) and circulating CA 15-3 (cCA 15-3)

	Healthy controls (n = 9)	pBC (n = 48)	mBC (n = 44)	p-value
Age (median, range)	63 (46–77)	56 (36–83)	65.5 (34–90)	0.21
Gender (F/M)	9/0	48/0	44/0	
Synchronous/metachronous disease	–	–	17/27	–
cCOL IV (ng/ml, median, range)	107.4 (92.9 – 149.1)	73.6 (44.6—187.7)	192 (89.1–1395.9)	< 0.0001
cCA 15-3 (IU/l, median, range)	13.8 (7.4–26.1)	17.8 (7.7 – 87.1)	127 (6 – 6000)*	< 0.0001

*Missing value. For one patient, cCA 15-3 could not be analysed in the mBC cohort

**Table 2:** Patient characteristics and values for circulating type IV collagen (cCOL IV) and circulating CA 15-3 (cCA 15-3) of metastatic breast cancer patients in relation to metastatic sites; liver metastases (LM), bone metastases (BM) and liver and bone metastases (LM + BM)

	LM (n = 5*)	BM (n = 26)	LM + BM (n = 13)	p-value
Age (median, range)	55 (50–74)	68 (34–91)	50 (34–84)	0.120
Gender (F/M)	5/0	26/0	13/0	
Primary tumour left (yes/no)	1/4	6/20	2/11	0.92
cCOL IV (ng/ml, median, range)	191.9 (98.4–302.2)	193.2 (89.1–568.9)	190.2 (118–1395.9)	0.483
cCA 15-3 (IU/l, median, range)	61.2 (19–783)*	142.5 (6–2246)	127 (13.9–6000)	0.619

* Missing value. For one patient with LM, cCA 15-3 could not be analysed

Regarding the tissue cohort, the 11 patients with BM were all female with BC metastases confirmed by pathologists; eight BM were located in the spine, two in the femur and two in the hipbone. The median age at the time of orthopaedic surgery was 73 years (range 52–84).

The 21 patients in the tissue cohort of LM were all female. The breast cancer origin of the liver metastases was confirmed by diagnostic pathology. The age distribution of the patients at surgery was as follows: 51 years (range 32–66).

### Metastatic BC patients have high levels of cCOL IV

**Fig. 1:**
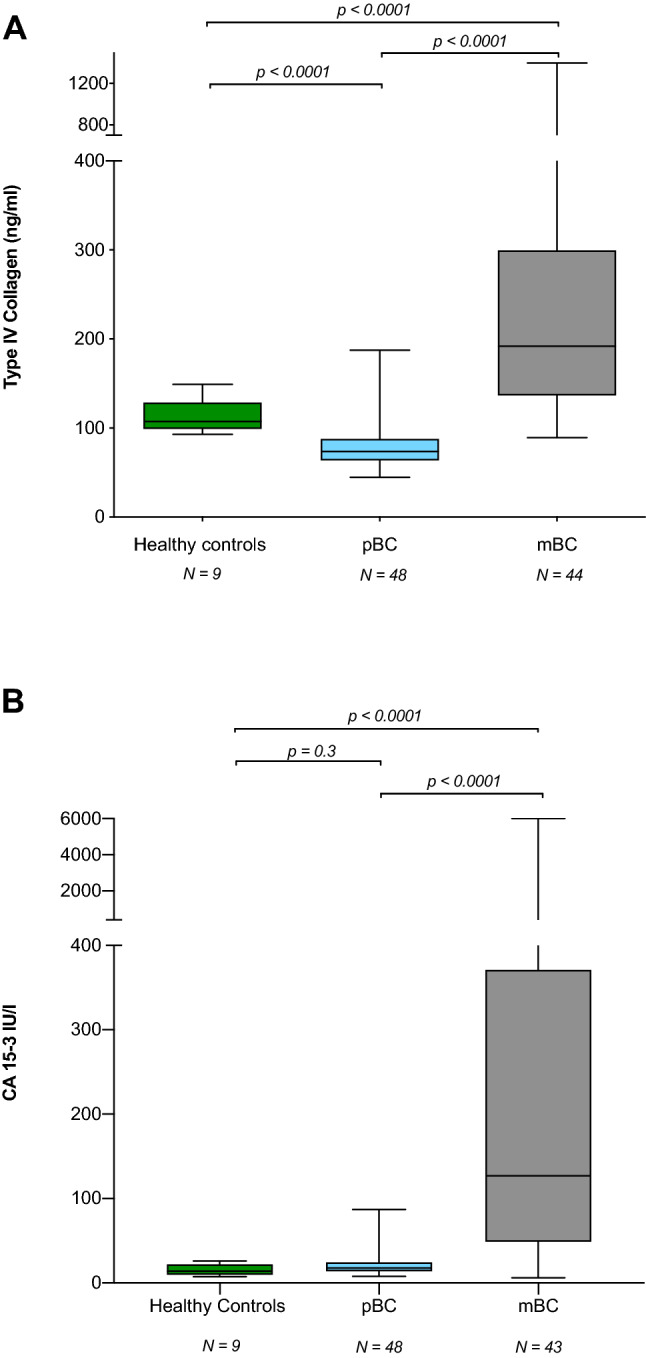
**a** Levels of circulating type IV collagen (cCOL IV) in healthy controls, patients with primary breast cancer (pBC) and metastatic BC (mBC). Patients with metastases had significantly higher levels of type IV collagen (192 ng/ml (89.1–1395.9)) compared with patients with primary BC (73.6 ng/ml (44.6–187.7)) (*p* < 0.0001)) and compared with healthy controls (107.4 ng/ml (92.9–149.1)) (*p* < 0.0001)). There was a significant difference between controls (107.4 ng/ml) and primary BC (73.6 ng/ml) (*p* < 0.0001). **b** Levels of circulating Ca 15-3 (cCA 15-3) in healthy controls, patients with primary breast cancer (pBC) and metastatic BC (mBC). For one patient with liver metastases, cCA 15-3 could not be analysed. Patients with metastases had significantly higher levels of CA 15-3 (127 IU/l (6 -6000)) compared with patients with primary BC (17.8 IU/l (7.7–87.1)) (*p* < 0.0001)) and compared with healthy controls (13.8 IU/l (7.4–26.1)) (*p* < 0.0001)). There was no significant difference between controls (13.8 IU/l) and primary BC (17.8 IU/l) (*p* = 0.3)

Higher levels of cCOL IV were found in control patients (n = 9) compared with pBC patients (n = 48) (107.4 ng/ml (92.9–149.1) *vs* 73.6 ng/ml (44.6–187.7) (*p* < 0.0001)) (Table 1, Fig. 1a). Significantly higher levels of cCOL IV were detected in patients with mBC (n = 44) compared with patients with pBC (n = 48) ((192 ng/ml (89.1–1395.9) *vs* 73.6 ng/ml (44.6–187.7)(*p* < 0.0001)) (Table 1, Fig. 1a) and in patients with mBC (n = 44) compared with control patients (n = 9) ((192 ng/ml (89.1–1395.9) *vs* 107.4 ng/ml (92.9–149.1) (*p* < 0.0001)) (Table 1, Fig. 1a). In patients with pBC, no differences in cCOL IV levels were seen between patients who subsequently developed a recurrence and those that did not. There was no significant difference in cCOL IV levels between ER + vs ER -, PR + vs PR- and HER2 + vs HER2—in metastatic BC patients (data not shown). There was no difference in cCOL IV in the mBC cohort between the nine patients with their primary tumour left versus the 35 patients where it was removed (data not shown).

### Metastatic BC patients have high levels of cCA 15-3

For one patient with LM, cCA 15-3 could not be analysed. There was a significant difference in the levels of cCA 15-3 between the pBC (n = 48) and mBC (n = 43) group (17.8 IU/l (7.7–87.1) *vs* 127 IU/l (6–6000) (*p* < 0.0001)) (Table 1, Fig. 1b). There were no significant differences in cCA 15-3 between control patients (n = 9) and pBC patients (n = 48) (*p* = 0.30) (Table 1, Fig. 1b). In patients with pBC, no differences in cCA 15-3 IV levels were seen between patients who subsequently developed a recurrence and those that did not. There was no significant difference in CA 15-3 levels between ER + vs ER -, PR + vs PR- and HER2 + vs HER2- in mBC patients (data not shown). There was no difference in cCA 15-3 in the mBC cohort between the nine patients with their primary tumour left versus the 35 patients where it was removed (data not shown).

### cCOL IV levels do not correlate with metastatic site

**Fig. 2:**
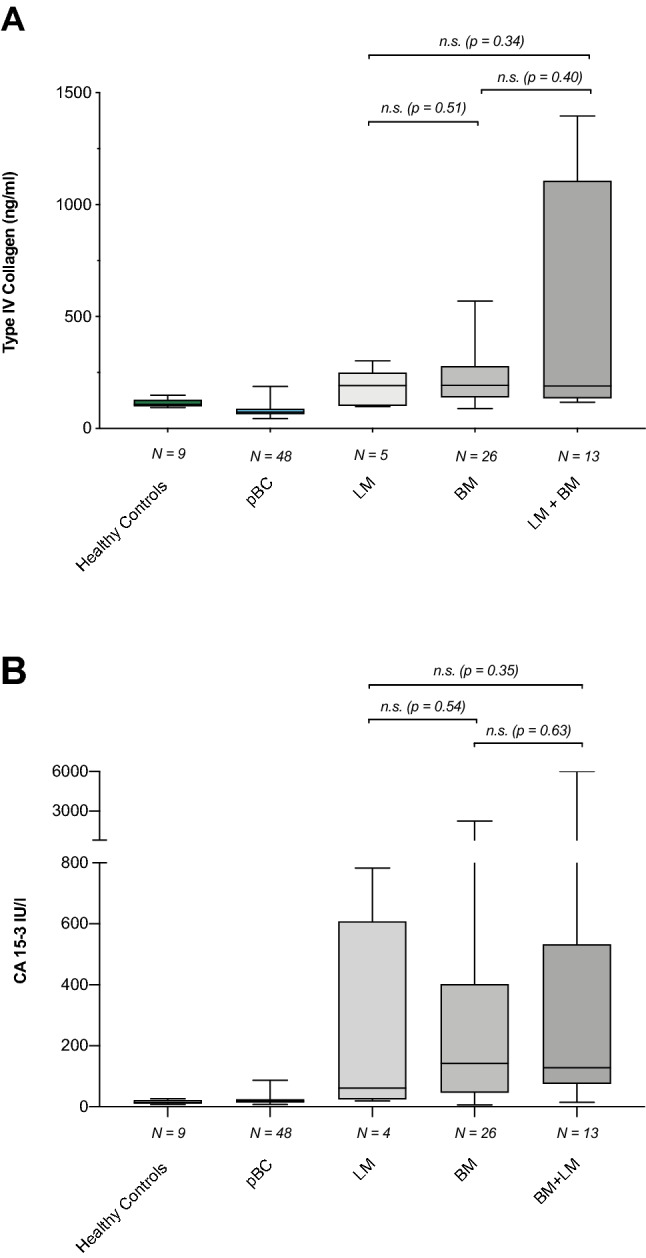
**a** Levels of circulating type IV collagen (cCOL IV) in healthy controls, patients with primary breast cancer (pBC) and metastatic BC with liver metastases (LM), bone metastases (BM) and both liver and bone metastases (LM + BM). There was no statistical difference between different metastatic sites (*p* = 0.483); LM (191.9 ng/ml (98.4–302-2)), BM (193.2 ng/ml (89.1–568.9)) and patients with both LM and BM (190.2 ng/ml (118–1395.9)). **b** Levels of circulating CA 15-3 (cCA 15-3) in healthy controls, patients with primary breast cancer (pBC) and metastatic BC with liver metastases (LM), bone metastases (BM) and both liver and bone metastases (LM + BM). For one patient with liver metastases, cCA 15-3 could not be analysed. There was no statistical difference between different metastatic sites (*p* = 0.619); LM (61.2 IU/l (19–783)), BM (142.5 IU/l (6–2246)) and patients with both LM and BM (127 IU/l (14–6000))

The level of cCOL IV was compared between patients with LM (n = 5) (191.9 ng/ml (98.4–302.2)), patients with BM (n = 26) (193.2 ng/ml (89.1–568.9)) and patients with both LM and BM (n = 13) (190.2 ng/ml (118–1395.9)). No statistical difference between the groups (*p* = 0.483) was found (Table 2, Fig. 2a).

### cCA 15-3 levels do not correlate with metastatic site

For one patient with LM, cCA 15-3 could not be analysed. The level of cCA 15-3 was compared between patients with LM (n = 4) (61.2 IU/l (19.0–783.0)), patients with BM (n = 26) (142.5 IU/l (6.0–2246)) and patients with both LM and BM (n = 13) (127.0 IU/l (13.9–6000)). No statistical difference between the groups (*p* = 0.619) was found (Table 2, Fig. 2b).

### High cCOL IV levels correlate with poorer survival for patients with mBC

**Fig. 3:**
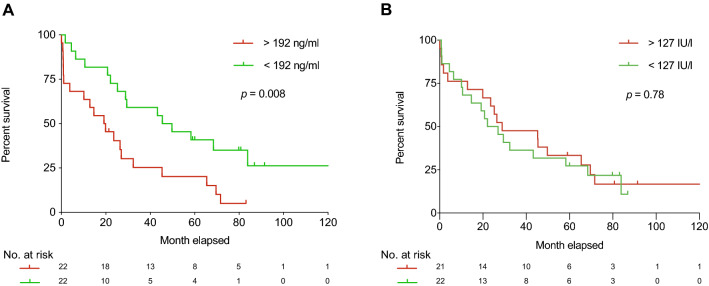
**a** Kaplan–Meier survival curve with a median follow-up time of 26.7 months. Patients with metastatic breast cancer (mBC) with high circulating levels of type IV collagen (> 192 ng/ml) had poorer survival compared with patients with low levels (< 192 ng/ml) (*p* = 0.008). **b** Kaplan–Meier curve with a median follow-up time of 26.7 months. Patients with metastatic breast cancer (mBC) were divided into groups of high and low circulating CA 15-3 based on median level. For one patient with liver metastases, cCA 15-3 could not be analysed. No differences in survival between the two groups were observed (*p* = 0.78)

A significant correlation between high cCOL IV levels and shorter survival times was observed (*p* = 0.008) (Fig. 3a) and the difference remained when adjusting for factors such as for ER and PR status and number of organs involved (p = 0.016). The median survival time in the group with cCOL IV levels (< 192 ng/ml) was 47.6 months compared with 19.5 months in the group with high cCOL IV levels (> 192 ng/ml). The median time for the development of metastatic disease in the mBC cohort was 25.6 months (range 0–291.6 months) and the median follow-up time was 26.4 months.

### Levels of cCA 15-3 do not correlate with survival for patients with mBC

No correlation between high cCA 15-3 levels and shorter survival time was observed (*p* = 0.78) (Fig. 3b). The median survival time in the group with low cCA 15-3 (< 127 IU/l) was 26.9 months compared with 27.7 months in the group with high cCA 15-3 levels (> 127 IU/l). The median time for the development of metastatic disease in the mBC cohort was 25.6 months (range 0–291.6 months) and the median follow-up time was 26.4 months.

### cCOL IV is superior to cCA 15-3 in detecting mBC

**Fig. 4:**
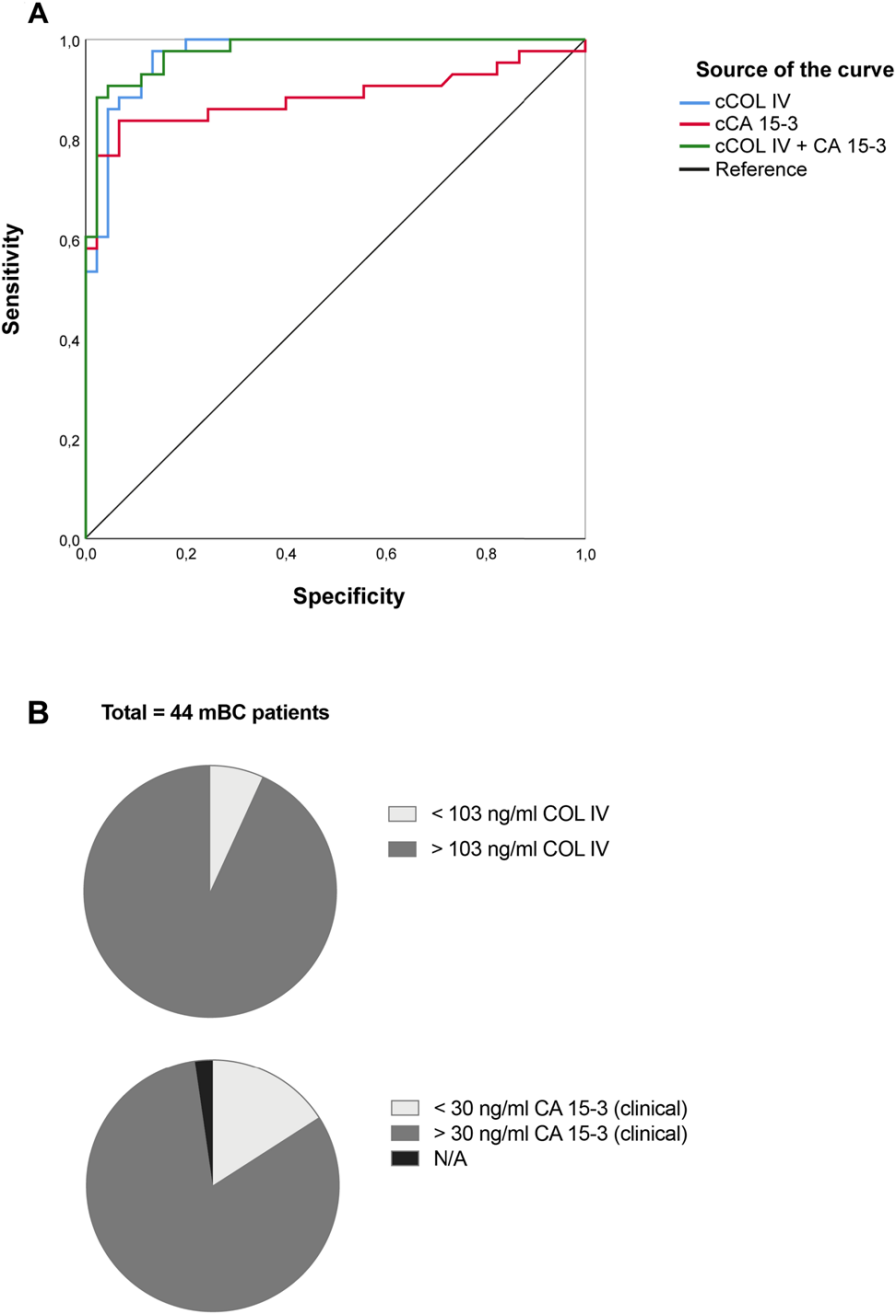
**a** ROC curve analysis of circulating type IV collagen (cCOL IV) and circulating CA 15-3 (cCA 15-3) and the combination of cCOL IV and cCA15-3 as biomarkers of metastatic BC-disease. The area under the curve (AUC) for cCOL IV (0.970) was significantly higher than for cCA 15-3 alone (0.884) (*p* = 0.0378). The AUC was significantly higher for the combination of both markers (0.976) compared with cCA 15-3 alone (0.884) (*p* = 0.0131). **b** Comparison of the number of mBC patients with cCOL IV levels above the calculated optimal cut-off and cCA 15-3 levels above both the calculated and the clinical cut-off. In 93% (n = 41) of mBC patients, the concentration of cCOL IV was higher than the calculated cut-off (> 103 ng/ml). In 84% (n = 36), the concentration of cCA 15-3 was higher than both the calculated and the clinical cut-off (> 30 IU/l *vs* > 35 IU/l)

For one patient with BC liver metastases, cCA 15-3 could not be analysed and this patient was omitted from the ROC analysis. The ROC analysis revealed that cCOL IV in this cohort is superior to cCA 15-3 in discriminating patients with mBC (n = 43) from patients with pBC (n = 48) (AUC = 0.970 *vs* 0.884, *p* = 0.0378) (Tables 3, 4, Fig. 4a). The ROC analysis furthermore showed that the combination of cCOL IV and cCA 15-3 is superior to cCA 15-3 at detecting mBC (*p* = 0.0131) (Tables 3, 4, Fig. 4a), but that the combination was not significantly superior to cCOL IV alone (*p* = 0.4443) (Tables 3, 4, Fig. 4a). For the two biomarkers, the optimal cut-off value for this cohort was determined by ROC curve analyses and the sensitivity and specificity are presented in Table 5. The calculated cut-off value for CA 15-3 in this cohort was higher than the clinically recommended level (35 IU/l vs. 30 IU/l). cCOL IV levels in mBC patients were more frequently elevated (n = 41, 93%) than cCA15-3 levels (n = 36, 84%) when using the calculated cCOL IV cut-off (103 ng/mL) compared with both the calculated and the clinically used cut-off for cCA 15-3 (> 30 IU/l n = 36 patients *vs* 35 > IU/l n = 36 patients) (Fig. 4b).

**Table 3:** Area under the curve (AUC) for circulating type IV collagen (cCOL IV), circulating CA 15-3 (cCA 15-3) and the combination of cCOL IV + cCA 15-3

	AUC	S.E	p-value
*Marker*			
cCOL IV	0.970	0.016	0.000
cCA 15-3	0.884	0.041	0.000
cCOL IV + cCA 15-3	0.976	0.013	0.000

**Table 4:** Comparison of the area under the curve (AUC) for circulating type IV collagen (cCOL IV) vs circulating CA 15-3 (cCA 15-3), cCOL IV vs the combination of both markers and cCA 15-3 vs the combination of both markers

	*p*-value
*Marker*	
cCOL IV *vs* cCA 15-3	0.038
cCOL IV *vs* combo	0.444
combo *vs* cCA 15-3	0.013

**Table 5:** Specificity, sensitivity and cut-off for the calculated values for circulating CA 15-3 (cCA 15-3) and circulating type IV collagen (cCOL IV) and the corresponding values for the clinically recommended cut-off for cCA 15-3

	I—specificity	Sensitivity	Cut-off (ng/ml, IU/l)
*Marker*			
cCA 15-3 calculated	6.5	83.7	35
cCA 15-3 clinical	8.7	83.7	30
cCOL IV calculated	10.6	93.2	103

### High expression of collagen IV in liver and bone metastases

**Fig. 5:**
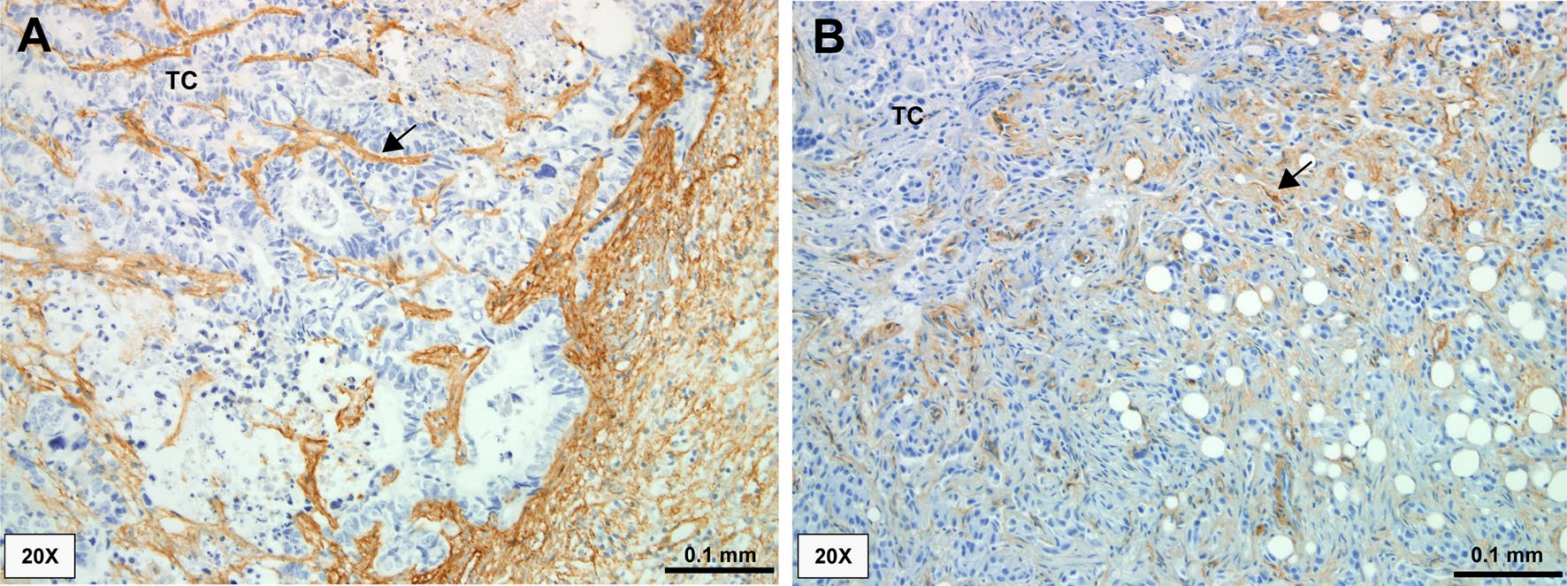
Tissue expression of collagen IV (COL IV) in breast cancer liver metastases (LM) (A) and in breast cancer bone metastases (BM)(B). In LM (**a**), COL IV (brown) is expressed intensely in the extracellular matrix (arrow) surrounding the tumour cells (TC) (blue). In BM (**b**), COL IV (brown) is expressed intensely in the extracellular matrix (arrow) surrounding the TC (blue). Magnification × 20

The tissue sections from 29 LM all showed a moderate-intense expression of COL IV in the metastases. Twenty-three LM were scored with high expression (2) and the remaining six LM as moderate expression (1). COL IV was expressed intensively in the ECM surrounding the tumour cells (TC). None of the patients showed negative staining for COL IV. COL IV was also detected in vascular BM and in the space of Disse in the normal part of the liver (Fig. 5a).

Nine of 13 paraffin sections of BC BM showed a high expression (2) and three sections a moderate expression (1) of COL IV (Fig. 5b). No sections were negative for COL IV. COL IV was found to be expressed in the ECM surrounding the TC, as well as in vascular basement membranes of the tissue.

## Discussion

A low-cost, non-invasive biomarker with high sensitivity to detect metastases at an early time point during the follow-up of patients with BC would have a high clinical value. A marker of this kind could, for example, make palliative treatment available at an earlier stage for mBC patients. Additionally, this marker could be used to monitor patients during palliative treatment, thereby replacing imaging. Today, no optimal circulating biomarkers for patients with mBC exist.

Our study shows that mBC patients have elevated plasma levels of cCOL IV compared with pBC patients and healthy controls and that high levels correlate with a poor prognosis, making it a potentially valuable biomarker of metastatic BC. We have also shown that cCOL IV actually outperforms cCA15-3 in detecting mBC disease.

Several studies have demonstrated the important role of COL IV in the metastatic process. An in-vitro study analysing the overexpression of COL IV in murine lung carcinoma cells shows that COL IV rescues cancer cells from anoikis and that the silencing of *COL IV* causes a reduction in the metastatic potential [27]. Moreover, COL IV is a stromal protein that is found in all the organs in the body and metastatic invasion involves the remodelling of the ECM, followed by tumour stroma production and the release of substances in the circulation. In pancreatic cancer patients, increased levels of COL IV are associated with a poor prognosis, but the levels of cCOL IV were able to predict a prognosis only when measured after removal of the primary tumour and therefore likely represents occult metastatic disease [25]. Similar results are seen in patients with colorectal cancer, where cCOL IV levels correlate with the hepatic tumour burden of CLM [19]. Our results showing that high cCOL IV predicts a poor prognosis could reflect tumour burden, but the prognostic relevance of high cCOL IV remained after adjusting for the number of organs involved with metastatic disease. However, we did not have radiological data to adjust for tumour size or tumour burden. It is very likely that the mBC patients had a larger tumour burden compared to the pBC patients. Based on the KM analysis (Fig. 3a) the curve representing patients with cCOL IV above the median of 192 ng/ml initially sloped quite fast compared to patients with cCOL IV below the median. This could imply that high cCOL IV levels could be an indicator of risk for death within the coming months.

This study shows that the site of metastasis did not affect circulating levels of CA 15-3 or COL IV and that the levels of cCOL IV seems to be a trait of the general metastatic process. Further, COL IV was highly expressed in the tissue of both LM and BM. Other studies indicate that an increase in the concentration of circulating biomarker depends on the type of metastases [14, 15]. Kokko et al. and Di Gioia et al. analysed sera from mBC patients with different metastatic sites and they reported that cCA 15-3 is elevated in all patients with LM but only in 40% and 47% respectively for BM. In addition, Kokko et al. showed that only 30% of patients with lung metastases had elevated levels [14, 15]. This is different from our results. However, their study design is not comparable to ours. Our study describes the median concentration of plasma cCOL IV and cCA 15-3 in patients with different metastatic sites, while Di Gioia et al. analysed sera and an elevation was defined as a 100% increase in the marker based on individual baseline values. Kokko et al. defined the elevation as a concentration above 40 IU/l. Neither Di Gioia et al. nor Kokko et al. studied cCOL IV. As a result, COL IV can still be a general marker of metastatic disease and, in order to confirm this, future studies should focus on analysing cCOL IV in larger cohorts with different primary cancers and metastatic sites. Furthermore, this study was of retrospective design and thus the metastatic sites were known at the time of the plasma sample for the mBC patients. It would be interesting to investigate whether cCOL IV could detect dormant metastatic disease, but this question would require a prospective design.

The patients with mBC in our study had elevated levels of both cCOL IV and cCA 15-3 and the combination of the biomarkers led to improved sensitivity in detecting mBC. These results corroborate other findings indicating that cCA 15-3 could be used in combination with other biomarkers to improve sensitivity and specificity [13, 14, 28]. However, in our study, the combination was not superior to cCOL IV alone.

The optimal cut-off for cCOL IV in detecting mBC in our cohort was 103 ng/ml, which is lower than that previously reported in publications; 115 ng/ml as the optimal cut-off for detecting CLM [21]. Additionally, the calculated optimal cut-off for cCA 15-3 was ≥ 35 IU/l, not the clinically used value of > 30 IU/l. There was, however, no difference in sensitivity between the two cut-offs and the difference in specificity was minimal. We also report a sensitivity of 83.7% for cCA 15-3 (cut-off > 30 IU/l). In comparison, Kokko et al. reported a sensitivity of only 36%, but the cut-off for cCA 15-3 was ≥ 40 IU/l[15]. This reflects the challenges of biomarker evaluation; different cohorts give rise to varying optimal cut-offs and varying sensitivity and specificity. For this reason, the potential biomarker must be validated in large patient cohorts in order to find a representative cut-off that can be used in the clinic.

This study is limited by the small number of patients in each cohort and the fact that the cohorts were derived from different hospitals. Additionally, the cohort with mBC patients was heterogeneous in terms of oncological treatment, tumour burden and follow-up time, which is, however, the reality of a metastatic disease. In our study, we found that the control patients had higher levels of cCOL IV than pBC patients. This could be due to the limited number of control patients, but it could also be due to variations in age between the groups [29].

Taken together, our results suggest that cCOL IV could be a valuable stroma-derived biomarker of mBC and support the view that not only the tumour cell compartment but also the stromal compartment should be taken into consideration when searching for new biomarkers.

## Conclusion

To summarise, this study indicates that cCOL IV is a potential non-invasive marker of metastatic disease in BC patients and that high circulating levels correlate with a poor prognosis. We also demonstrate that tissue from patients with LM and BM has a high expression of COL IV in the extracellular matrix surrounding the TC. In our study, cCOL IV actually outperformed cCA 15-3 in detecting mBC patients. Our results support the hypothesis that a stromal non-invasive biomarker such as type IV collagen can be used to detect mBC.

## Supplementary Information

Below is the link to the electronic supplementary material.

Electronic supplementary material 1 (DOCX 14 kb)

## Data Availability

The data presented in this study can be shared in response to a reasonable request to the corresponding author.
