# Supplementary material for: Type IV collagen as a potential biomarker of metastatic breast cancer
Source: Clin Exp Metastasis. 2021 Mar 3;38(2):175–85. doi: 10.1007/s10585-021-10082-2 (PMC7987628; doi:10.1007/s10585-021-10082-2)
Supplement: Supplementary file 1 — Electronic supplementary material 1 (DOCX 14 kb) [file 10585_2021_10082_MOESM1_ESM.docx]

**Supplementary table 1**

Additional patient characteristics for patients with primary breast cancer (pBC) and metastatic breast cancer (mBC). All the presented data are based on the primary breast cancer tumour, except for age that is presented at either the time of diagnosis of the primary breast cancer (pBC cohort) or the time of the diagnosis of metastatic disease (mBC cohort).

**Supplementary Table 1**

|  | **pBC** (n=48) | **mBC** (n =44) |
| --- | --- | --- |
| **Age**  ≤ 50 51-60 ≥ 61 | 12 18 18 | 9  10  25 |
| **Tumour size (mm)**  ≤ 20 21-50 ≥ 50 | 24 21 3 | NA* |
| **Nodal status**  Positive Negative | 10 38 | NA* |
| **Tumour grade**  1 2 3 Not known | 0 16 32 | 4 24 14 2 |
| **IDC mixed with DCIS**  Yes No | 34 14 | NA* |
| **Oestrogen receptor**  Positive Negative | 30 18 | 40 4 |
| **Progesterone receptor**  Positive Negative | 30 18 | 36 8 |
| **HER2 receptor**  Positive Negative Not known | 13 35 | 6 37 1 |
| **Ki67 status**  Low (<20%) High (>20%) | 35 13 | NA* |
| **Molecular subtype**  Luminal A Luminal B (HER2-) Luminal B (HER2+) HER2 positive Triple negative Not known | 9 17 4 9 9  0 | 19 13 6 0  4 2 |

Abbreviations: pBC: primary breast cancer patients; mBC: metastatic breast cancer patients;
IDC: invasive ductal cancer; DCIS: ductal carcinoma in situ. NA* data not available.
